# BDJ Open—4 years on

**DOI:** 10.1038/s41405-019-0027-7

**Published:** 2020-01-08

**Authors:** Jonathan Lewney

**Affiliations:** British Dental Association, London, UK

**Keywords:** Health care, Dentistry

BDJ Open ‘opened’ in 2015 and as editor I have had the genuine pleasure of seeing a huge range of articles submitted and published for anyone with an internet connection to read.

The benefits of open access publishing are relatively easy to describe and quantify:

- a vastly expanded scope (as no confines of publishing space).
- relatively short publication timeframes (no need to wait for a space in the print copy).
- and the obvious advantage that anyone can access an open access article without the need for subscriptions or log-ins.

The benefits to readers, and the whole dental/academic community, of this form of publishing are perhaps less easy to quantify but are where the real excitement in an open access journal lies. Subscription journals naturally need to publish articles targeted to their subscribers, and the space constraints of a printed journal compound this. The range of study topics and methods submitted and published in BDJ Open has exceeded expectations. To date we have published:

Randomised Control Trials:

Microstream capnography during conscious sedation with midazolam for oral surgery: a randomised controlled trial

Service evaluations:

A service evaluation of the “Open Wide and Step Inside” school oral health programme

Economic analysis:

Pharmacoeconomic analysis of antibiotic therapy in maxillofacial surgery

And some exciting developments from researchers in industry:

Stain control with two experimental dentin hypersensitivity toothpastes containing spherical silica: a randomised, early-phase development study

Reviewers sometimes recommend rejection as the subject matter is ‘not of interest’ or there is ‘no reason to publish this’. The beauty of open access publishing is that, provided an article is of acceptable quality, there is really no reason not to accept it. It’s up to the world to decide if they want to read the articles. A journal with a narrower scope and more space constraints might find it difficult to justify publications on the experience of dental professionals from anywhere across the globe such as:

Public Dental Service personnel facing a major health care reform in Finland

Fortitude and resilience in service of the population: a case study of dental professionals striving for health in Sierra Leone

Open access allows these authors to publish in the BDJ portfolio for anyone to compare their practices and conditions with the worldwide dental community. This international element is a particular benefit of open access publishing. Techniques and instruments, which wouldn’t be used in higher income countries due to higher cost alternatives, might be of interest to dentists in lower income countries. BDJ Open makes this possible. This includes research instruments such as the Oral Health Impact Profile:

Oral health-related quality of life in patients with upper gastrointestinal and hepatic disorders in Pakistan: validation of the Oral Health Impact Profile-14 in the Urdu language.

Authors from low income countries can also apply for reduced fees or waivers, meaning anyone can learn about dental students in Nepal and their knowledge of implants:

Knowledge and perception about dental implants among undergraduate dental students.

In terms of the quality assurance of the publishing process, it’s the same process as the print BDJ, often the same reviewers, and the same Editor-in-Chief (Stephen Hancocks) who has the final say. But is the quality of the actual research the same? With open access publishing anyone at all is free to answer that question. Our brief to reviewers is as follows:

“To be published in BDJ Open, a paper must be scientifically valid and technically sound in methodology.”

When open access publishing began there was a common concern that open access journals could offer a ‘you pay, we publish’ service. Whilst any topics related to oral and dental health are within scope, the peer review process means that this has been far from true for BDJ Open. Importantly, challenges to articles can be dealt with through published correspondence for anyone to access. The published correspondence on the use of LED light curing instruments in lower income countries exemplifies many of the points raised in this editorial.

Reply to: Relevance of investigating light transmittance through red protective shields in dentistry

It’s been exciting to publish in more niche areas (providing an interesting challenge in finding reviewers) such as a genetics paper examining links between hypodontia and colorectal cancer:

Variations in *AXIN2* predict risk and prognosis of colorectal cancer

We were also really interested to publish research on a situation lots of dental professionals will recognise but which has not, to our knowledge, been published elsewhere:

What reward does a child prefer for behaving well at the dentist?

In summary, BDJ Open continues to bring oral health research to as wide an audience as possible. The breadth and depth of research published in BDJ Open to date shows that authors across the world value the opportunity to publish in a BDJ journal. These are exciting times for publishing in general and we are very proud of the contribution BDJ Open is making to the global dental community.

For anyone interested in publishing in BDJ Open or reviewing submissions to BDJ Open please see the author and referee instructions: https://www.nature.com/bdjopen/authors-and-referees or contact us at  bdjopen@nature.com.

